# Effects of face masks on acoustic analysis and speech perception: Implications for peri-pandemic protocolsa)

**DOI:** 10.1121/10.0002873

**Published:** 2020-12-10

**Authors:** Michelle Magee, Courtney Lewis, Gustavo Noffs, Hannah Reece, Jess C. S. Chan, Charissa J. Zaga, Camille Paynter, Olga Birchall, Sandra Rojas Azocar, Angela Ediriweera, Katherine Kenyon, Marja W. Caverlé, Benjamin G. Schultz, Adam P. Vogel

**Affiliations:** Centre for Neuroscience of Speech, The University of Melbourne, 550 Swanston Street, Carlton, VIC 3053, Australia

## Abstract

Wearing face masks (alongside physical distancing) provides some protection against infection from COVID-19. Face masks can also change how people communicate and subsequently affect speech signal quality. This study investigated how three common face mask types (N95, surgical, and cloth) affected acoustic analysis of speech and perceived intelligibility in healthy subjects. Acoustic measures of timing, frequency, perturbation, and power spectral density were measured. Speech intelligibility and word and sentence accuracy were also examined using the Assessment of Intelligibility of Dysarthric Speech. Mask type impacted the power distribution in frequencies above 3 kHz for the N95 mask, and above 5 kHz in surgical and cloth masks. Measures of timing and spectral tilt mainly differed with N95 mask use. Cepstral and harmonics to noise ratios remained unchanged across mask type. No differences were observed across conditions for word or sentence intelligibility measures; however, accuracy of word and sentence translations were affected by all masks. Data presented in this study show that face masks change the speech signal, but some specific acoustic features remain largely unaffected (e.g., measures of voice quality) irrespective of mask type. Outcomes have bearing on how future speech studies are run when personal protective equipment is worn.

## I. INTRODUCTION

Face masks (alongside physical distancing) provide some protection against infection from Coronavirus disease (COVID-19) (Chu *et al.*, 2020). Their use in public spaces and healthcare settings is either recommended or mandatory in many jurisdictions internationally. In the United States, the Center for Disease Control (CDC, 2020) recommends mask use to minimize droplet dispersion and aerosolization of the virus (Bahl *et al.*, 2020). Clinical trials and healthcare settings continue to assess speech production, which generates respiratory droplets while unrestricted exposure increases the likelihood of disease contraction (Stadnytskyi *et al.*, 2020). Risk of transmission increases through behaviors common in many speech assessment tasks including continuous and loud speech (Asadi *et al.*, 2019). At the same time, acknowledgement of the necessity of personal protective equipment (PPE) to minimize virus transmission has increased internationally (Asadi *et al.*, 2019; Stadnytskyi *et al.*, 2020; Zaga *et al.*, 2020). Masks, however, alter the speech signal with downstream effects on intelligibility of a speaker. The use of personal protective equipment poses some unique challenges for speech assessment.

Most masks prevent visual access to the speaker's lips and create a barrier during communication. This in itself can hinder speech perception, especially in noisy environments or when the listener has a hearing impairment (Hampton *et al.*, 2020). Masks can muffle speech sounds, especially higher frequencies that can aid the differentiation of similar sounds. The acoustic effect of a speaker wearing a face mask is equivalent to the listener having a slight high-frequency hearing loss (Corey *et al.*, 2020). The type of mask worn may uniquely affect acoustic and speech perception, as mask types vary in their composition and how they are designed to sit on the wearer's face. The three most common mask types for preventing disease transmission are cloth, surgical, and N95 filtering facepiece respirator (N95 mask). Cloth masks are often 2-ply and made from a single fabric type (e.g., cotton). The tightness of the fabric weave and mask fit vary widely. Surgical masks are commonly 3-ply nonwoven fabric with a water-resistant outer layer, filter middle layer, and water-absorbing inner lay. Surgical masks fit loosely on the face with air able to escape from the sides. N95 masks are similar in composition to surgical masks with the main differences being a higher filtration rate and a tight seal around the wearer's face, preventing air escape (O'Dowd *et al.*, 2020).

We evaluated the impact wearing a mask has on acoustic output and speech perception. We examined how different face mask types (surgical, cloth, and N95), in combination with microphone variations (headset vs tabletop), affect speech recordings and perceived intelligibility. We hypothesized the acoustic output would be impacted based on the composition/thickness of masks while perceived intelligibility will remain largely unaffected.

## II. METHODS

Overall, seven subjects (aged 28.1 ± 6.0 years, range 21–39; four males, three females), were included in the study. All speakers were English speaking with no dysphonia, cognitive, or neurological impairments. One male (15 years since exposed to English) and female (26 years since exposed to English) were subsequent bilinguals and reported English as their second language.

### A. Speech Acquisition and feature extraction for acoustic output

**FIG. 1.**
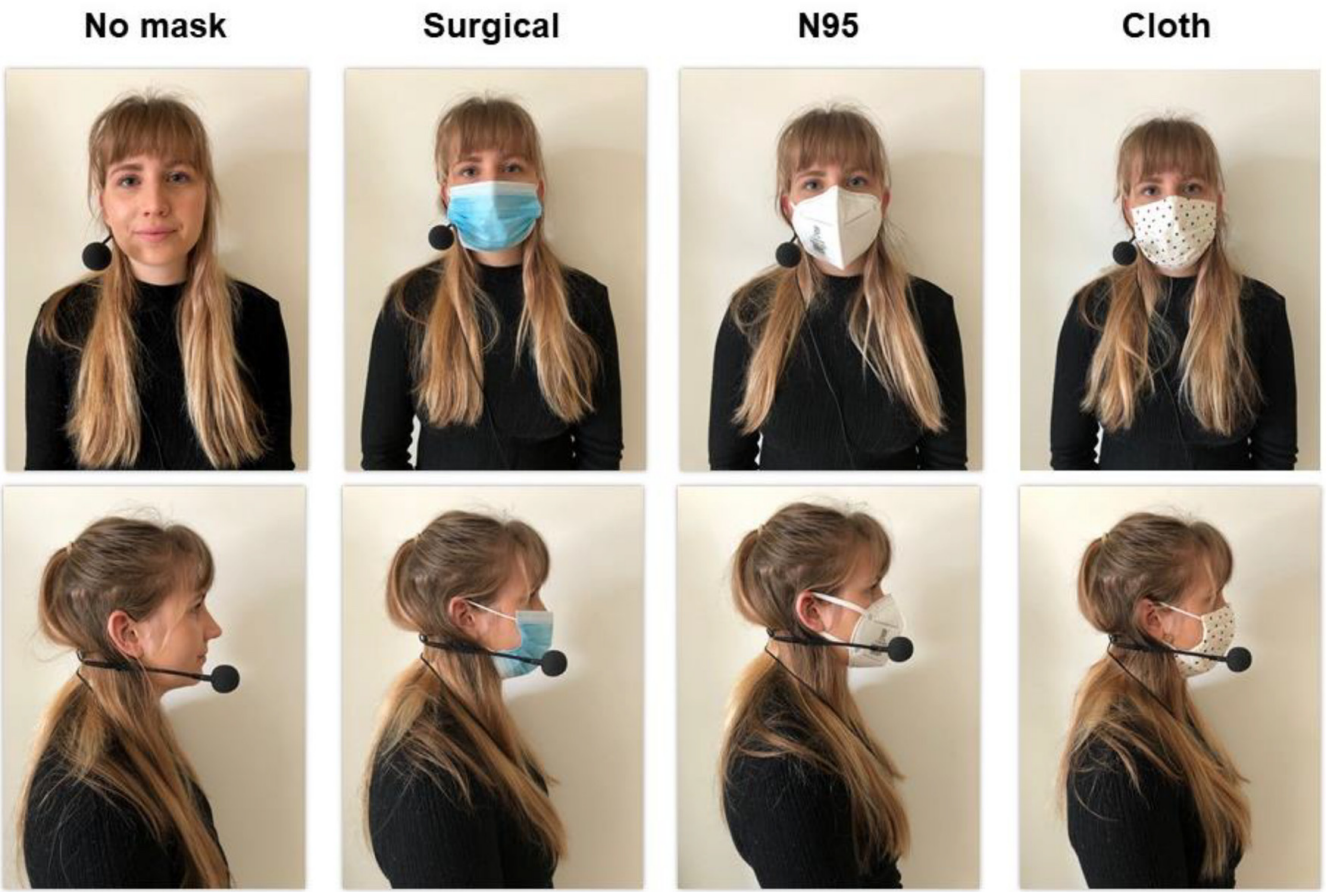
(Color online) Mask conditions.

Four subjects (aged 29.3 ± 6.0 years; two males, two females) completed a speech battery consisting of sustaining an open vowel /aː/ for approximately six seconds, reproduced ten times and reading a phonetically balanced text, the Grandfather Passage (Van Riper, 1963), reproduced five times. The speech battery was repeated under four conditions using inter-subject counter balancing. Conditions included (1) no mask, (2) standard surgical mask (regulated under 21 CFR 878.4040), (3) cloth mask (2-layered cotton), and (4) N95 mask (electrostatic non-woven polypropylene fiber containing a filtration layer) (Fig. 1). Subjects were instructed to speak in a natural manner at a comfortable pitch and pace.

Speech samples were simultaneously recorded using two standardized methods: (1) Using a head-mounted cardioid condenser microphone (AKG520, Harman International, Stamford, CT) positioned two inches from the corner of the subject's mouth (minimum sensitivity of −43 dB, near flat frequency response) and coupled with a QUAD-CAPTURE USB 2.0 Audio Interface (Roland Corporation, Shizuoka, Japan) connected to a laptop computer, and (2) Using a Blue Yeti (Blue Microphones, Westlake Village, CA) tabletop microphone (sensitivity 4.5 mV/Pa) connected to a laptop computer. The microphone was positioned 5 feet from the subject to simulate physical distancing measures. Standardization of the recording environment was achieved by recording in the absence of traffic, electrical, appliance, or other background noise. All recordings were sampled at 44.1 kHz with 32-bit quantization. Each recording produced was ∼40 min in length (per subject).

Audio files were screened for deviations and synchronized between microphones to ensure uniformity of length. Acoustic measures of timing, frequency, power spectral density (PSD), and perturbation extracted from sustained vowel and reading tasks using Praat software (Boersma, 2001). Acoustic measures were specifically chosen to estimate the overall loss of speech-sound intensity or attenuation effect (speech intensity prominence), frequency-specific loss of intensity or filtering effect (PSD, center-of-gravity, COG), and possible consequences of filtering on commonly used measures of voice quality (harmonic-to-noise ratio and cepstral peak prominence). We additionally included commonly used measures which are likely independent of attenuation or filtering effects, namely, fundamental frequency and speech-silence analysis on the time domain. Measures of timing (detection of silence-speech and speech-silence transitions) were extracted using an energy threshold on the time domain (Rosen *et al.*, 2010; Vogel *et al.*, 2017). The threshold was set to 65% of the 95th percentile, with minimum silence length set to 20 ms and minimum speech length to 30 ms. Fundamental frequency was calculated through autocorrelation within a restricted range (70–250 Hz for males, 100–300 Hz for females) (Vogel *et al.*, 2009). The analysis window was 43 and 30 ms, respectively, and window shift fixed at 10 ms. The maximum number of formants was set at 5 with a maximum of 5500 Hz for formant detection. All other parameters were maintained at default software settings. The PSD (dB/kHz relative 2 × 10^−5 ^Pa) in the long-term average spectrum was extracted from the reading task to information on how “each frequency” contributes to the total sound power. CoG (in Hz) is defined as the mean frequency that divides the power spectrum in equal halves was calculated from the power spectrum.

The intensity of background noise (floor) was determined as equal to the average intensity during the quietest three seconds of each files (i.e., in the absence of vocalization). Floor intensity was subtracted from the average intensity (during vocalization) for each task (vowel and reading) to determine the speech intensity prominence. Features of interest included cepstral peak prominence smoothed (CPPS), harmonic-to-noise ratio (HNR), local jitter, and shimmer for the sustained vowel, and average and standard deviation of pause length for the reading task.

### B. Speech acquisition and analysis for speech intelligibility

Single word and sentence intelligibility were evaluated in five subjects (aged 29.3 ± 7.1 years; two males, two females) using the Assessment of Intelligibility of Dysarthria Speech (ASSIDS) (Yorkston and Beukelman, 1984). The assessment involved subjects reading 50 randomly selected one- or two-syllable words and 22 randomly selected sentences, ranging in length from 5 to 15 words. Subjects were instructed to speak in a natural manner at a comfortable pitch and pace. ASSIDS was assessed under four conditions (inter-subject counter balance of no mask, surgical, N95, and cloth masks). Each subject's responses were audio-recorded using the tabletop microphone (sensitivity 4.5 mV/Pa) positioned 5 feet from the subject (recording produced was ∼20 min in length per subject).

Each recording was then blinded to mask condition and transcribed by five independent listeners (aged 37.5 ± 9.0 years; one male, four females; two listeners reported English as their second language). Listeners were asked to transcribe the word/sentence the subject was trying to say. Transcriptions were the collated and scored for each condition, with a total score of 50 for words and 220 for sentences. Scores were then converted to percentage to establish intelligibility of single words (ASSIDS words intelligibility) and sentences (ASSIDS intelligibility sentences) for each mask condition. Inter-rater reliability was calculated using Fleiss multi-rater Kappa. Fleiss' kappa showed a high degree of agreement between listeners on single words (*κ* = 0.88, *p* < 0.001) and sentences (*κ* = 0.74, *p* < 0.001).

### C. Statistical analysis

To understand the effect of masks on perceived intelligibility (ASSIDS words and sentences intelligibility) and acoustic parameters, a linear mixed-effects model analysis with restricted maximum likelihood estimation was applied. Mask type was modeled as a fixed factor, and subject, and order of mask condition as a random factor. Bonferroni corrected *planned* comparisons were conducted to determine differences in mask type (surgical, N95, and cloth) compared to no mask.

To investigate the effect of masks on the PSD, the interaction between mask condition × frequency band (1 kHz bins) was investigated using a linear mixed-effects model analysis with restricted maximum likelihood estimation. Subject, and order of mask condition as a random factor. Bonferroni corrected *planned* comparisons were made for each 1 kHz frequency bin (1–10 kHz) to determine differences between masks types compared to no mask. SPSS Statistics was used for these statistical analyses (IBM SPSS Version 26.0).

To assess differences in single word and sentence accuracy, a generalized linear mixed-effects model was applied to binomial accuracy data (0:incorrect word/sentence, 1:correct word/sentence) with mask type (4; no mask, surgical, N95, and cloth) and phrase (2; single word, sentence) as fixed factor, with subject and stimulus identity as random factors using the *lme4*, *afex*, and *effectsize* libraries. Planned comparisons were performed using Tukey's Honestly Significant Difference to determine differences in mask type using the *multcomp* library R 4.0.2 was used for this statistical analysis (R Development Core Team, 2020).

## III. RESULTS

### A. Speech intelligibility outcomes

**FIG. 2.**
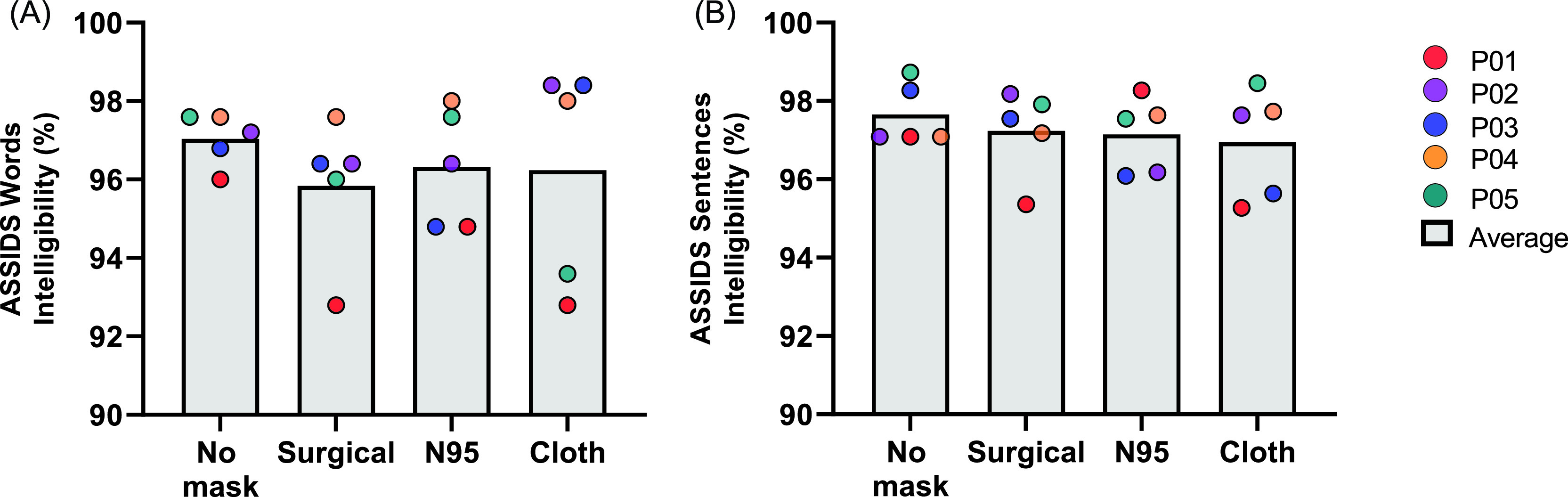
(Color online) Average speech intelligibility based on mask condition using the ASSIDS.

ASSIDS single word and sentence intelligibility varied between the speakers and across mask conditions (Table I). Anecdotally, there was greater variability in ASSIDS single word intelligibility [range 82%–100%; Fig. 2(a)], than ASSIDS sentences intelligibility [range 91.8%–100%; Fig. 2(b)]. There was no significant effect of masks on intelligibility for either ASSIDS single words (*F*_3,34.70_ = 0.60, *p* = 0.621) or ASSIDS sentences (*F*_3,34.58_ = 0.73, *p* = 0.542).

**TABLE I.** Single word and sentence intelligibility for each subject based on mask condition using the ASSIDS. Values represent mean ± standard deviation of five independent listeners.

	No mask	Surgical	N95	Cloth
**ASSIDS word intelligibility (%)**				
P01	96.00 ± 5.83	92.80 ± 4.60	94.80 ± 2.28	92.80 ± 6.57
P02	97.20 ± 2.68	96.40 ± 2.61	96.40 ± 2.97	98.40 ± 1.67
P03	96.80 ± 2.28	96.40 ± 2.97	94.80 ± 1.10	98.40 ± 2.61
P04	97.60 ± 2.61	97.60 ± 1.67	98.00 ± 2.00	98.00 ± 2.00
P05	97.60 ± 1.67	96.00 ± 2.83	97.60 ± 1.67	93.60 ± 2.97
**ASSIDS sentence intelligibility (%)**				
P01	97.09 ± 0.52	95.36 ± 1.49	98.27 ± 1.34	95.27 ± 2.17
P02	97.09 ± 1.94	98.18 ± 1.70	96.18 ± 1.46	97.64 ± 1.71
P03	98.27 ± 1.49	97.55 ± 0.69	96.09 ± 0.94	95.64 ± 2.19
P04	97.09 ± 2.62	97.18 ± 1.26	97.64 ± 1.22	97.73 ± 1.07
P05	98.73 ± 0.99	97.91 ± 0.41	97.55 ± 0.76	98.45 ± 0.52

Further investigation revealed that identification accuracy of single word and sentences significantly varied between phrase types (*χ*^2^ = 94.98, *p <* 0.001, Cramer's *V* = 0.11) and across mask conditions (*χ*^2^ = 15.03, *p* = 0.002, Cramer's *V* = 0.05), although the interaction was not significant (*χ*^2^ = 1.32, *p* = 0.73, Cramer's *V* = 0.01). Pairwise comparisons revealed decreased accuracy for speakers wearing a surgical (*p* = 0.029), N95 (*p* = 0.014), or cloth mask (*p* = 0.012) compared to no mask; there were no significant differences between masks (*ps* > 0.99).

### B. PSD extracted from reading task under different mask conditions

**FIG. 3.**
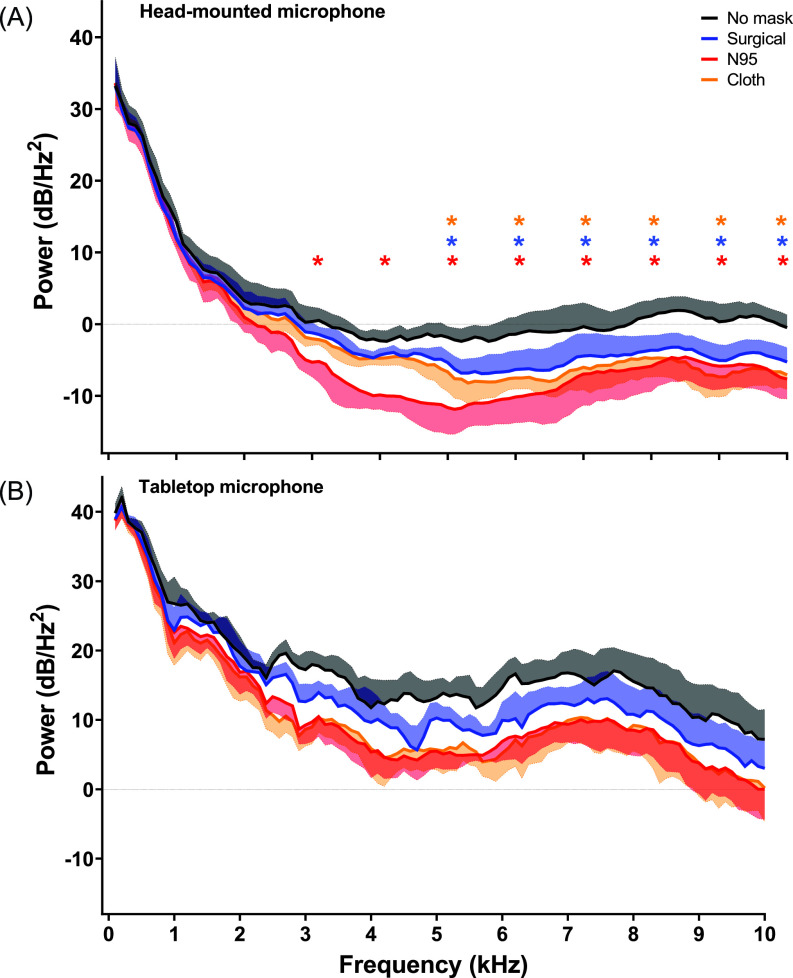
(Color online) PSD extracted from reading task under different mask conditions. Mean power spectra density displayed between 1 and 10 kHz based on mask type. Shaded areas represent the standard error of mean. *p ≤ 0.05 no mask vs mask type at each frequency bin. Red stars denote significant differences between no mask and N95, blue stars denote significant differences between no mask and surgical masks, while orange stars denote significant differences between no mask and N95.

Frequency bands were collapsed into 1 kHz bins to explore differences in PSD between mask type. There was a Mask × frequency band interaction effect (*F*_27_ _755_ = 2.50, *p* = 0.006). *Post hoc* comparisons showed power (dB/Hz^2^) was significantly lower between 3 and 10 kHz for the N95 mask (*p* < 0.001) and 5–10 kHz for both the surgical (*p* < 0.001) and cloth masks (*p* < 0.001) when compared to no mask on recordings made using the head-mounted microphone [Fig. 3(a)]. No significant differences were observed between mask conditions on recordings made using the tabletop microphone [*F*_27_ _757_ = 1.41, *p =* 0.082; Fig. 3(b)].

### C. Acoustic parameters extracted from sustained vowel and reading tasks

For recordings produced with the head-mounted microphone, there was a significant effect of masks for mean pause length (*F*_3,8.97_ = 3.88, *p* = 0.05), percentage of pauses (*F*_3,8.40_ = 7.36, *p* = 0.01), and spectral tilt (*F*_3,8.98_ =13.62, *p* = 0.001) extracted from the reading task. *Post hoc* comparisons showed that recordings produced with the N95 mask increased percentage of pauses (*p* = 0.023) (Table II). Spectral tilt was lower in recordings produced with the surgical (*p* = 0.016) and N95 masks (*p* = 0.001). For recordings produced with the tabletop microphone, there was a significant effect of mask type for percentage of pauses (*F*_3,7.87_ = 8.17, *p* = 0.008), and spectral tilt (*F*_3,8.39_ = 15.43, *p* = 0.001) (Table II). *Post hoc* comparisons revealed that the N95 and cloth masks yielded higher percentage of pauses (N95 *p* = 0.022; Cloth *p* = 0.029) no mask. As with the head-mounted microphone, recordings produced with the tabletop microphone yielded lower spectral tilt values with both the surgical (*p* = 0.006) and N95 masks (*p* = 0.002). No significant differences were observed in acoustic parameters extracted from the sustained vowel recorded using either the head-mounted or tabletop microphone.

**TABLE II.** Acoustic parameters extracted from the reading task recordings produced by the head-mounted and tabletop microphones under different mask types. *p ≤ 0.05, ^**^p ≤ 0.01, ^***^p ≤ 0.001. Values represent mean ± standard deviation. CI = Confidence Interval.

						Mean difference (95% CI)		
	No mask	Surgical	N95	Cloth	*F*	No mask vs surgical	No mask vs N95	No mask vs cloth
**Head-mounted microphone**								
Mean pause length (seconds)	0.24 ± 0.07	0.24 ± 0.08	0.28 ± 0.10	0.26 ± 0.10	3.88^*^	0.008 (−0.053, 0.036)	0.032 (−0.012, −0.077)	0.019 (−0.025, 0.063)
Variability of pause length	0.36 ± 0.09	0.38 ± 0.14	0.44 ± 0.16	0.43 ± 0.17	3.14			
Percent of pauses (%)	30.3 ± 3.88	31.74 ± 2.56	35.42 ± 3.08	34.94 ± 2.76	7.36^**^	1.00 (−3.23, 5.22)	4.91^*^ (0.66, 9.17)	4.25 (−0.02, 8.52)
								
Spectral tilt (dB)	−21.4 ± 3.32	−16.73 ± 1.84	−14.5 ± 3.06	−18.86 ± 3.65	13.62^***^	4.65^*^ (0.83, 8.47)	6.92^***^ (3.10, 10.74)	2.49 (−1.33, 6.31)
Mean intensity (dB)	63.61 ± 3.04	63.04 ± 3.35	63.27 ± 3.75	62.05 ± 3.05	1.50			
Intensity prominence	42.86 ± 2.03	40.66 ± 3.07	41.68 ± 2.18	40.01 ± 2.73	2.68			
*p*95 Intensity	64.37 ± 3.01	63.83 ± 3.37	64.05 ± 3.72	62.97 ± 2.97	1.19			
CPPS	19.40 ± 2.89	20.58 ± 1.76	20.8 ± 2.57	20.31 ± 2.75	2.21			
HNR	24.68 ± 3.45	25.48 ± 3.23	25.84 ± 5.09	26.56 ± 3.78	1.41			
*f*0 mean (Hz)	155.42 ± 63.82	155.09 ± 66.08	154.1 ± 63.90	162.25 ± 60.69	0.92			
*f*0 CoV (%)	0.76 ± 0.07	0.72 ± 0.08	0.63 ± 0.08	0.72 ± 0.09	2.60			
Jitter (%)	0.31 ± 0.07	0.36 ± 0.09	0.31 ± 0.07	0.34 ± 0.09	1.45			
Shimmer (%)	1.51 ± 0.23	1.55 ± 0.16	1.64 ± 0.5	1.51 ± 0.24	0.49			
**Tabletop microphone**								
Mean pause length (seconds)	0.38 ± 0.16	0.40 ± 0.17	0.41 ± 0.18	0.42 ± 0.20	0.80			
Variability of pause length	0.41 ± 0.13	0.46 ± 0.17	0.50 ± 0.17	0.50 ± 0.19	3.29			
Percent of pauses (%)	25.37 ± 4.84	26.25 ± 4.50	29.04 ± 4.47	28.91 ± 5.56	8.17^**^	0.80 (−2.21, 3.81)	3.54^*^ (0.50, 6.57)	3.39* (0.34, 6.44)
Spectral tilt (dB)	−30.82 ± 1.43	−24.78 ± 1.82	−23.59 ± 4.09	−29.32 ± 4.96	15.43^***^	6.59^**^ (2.03, 11.15)	7.65^**^ (3.09, 12.21)	1.80 (−2.76, 6.35)
Mean intensity (dB)	71.54 ± 3.89	71.73 ± 4.34	71.85 ± 4.31	72.26 ± 2.78	0.12			
Intensity prominence	37.09 ± 3.91	36.67 ± 4.35	36.94 ± 4.5	37.57 ± 3.12	0.22			
*p*95 Intensity	72.66 ± 3.76	72.87 ± 4.3	72.95 ± 4.37	73.52 ± 2.84	0.19			
CPPS	19.52 ± 2.74	19.16 ± 1.87	19.99 ± 2.19	19.34 ± 2.1	0.52			
HNR	20.30 ± 3.66	19.11 ± 3.25	21.88 ± 3.77	21.37 ± 2.16	1.19			
*f*0 mean (Hz)	155.80 ± 63.25	155.4 ± 64.64	156.4 ± 61.32	169.77 ± 45.03	0.92			
*f*0 CoV (%)	0.71 ± 0.09	0.77 ± 0.08	0.65 ± 0.08	0.65 ± 0.06	2.41			
Jitter (%)	0.32 ± 0.06	0.36 ± 0.11	0.31 ± 0.08	0.32 ± 0.06	0.98			

## IV. DISCUSSION

The type of mask affected the speech signal. We observed significant differences in acoustic power distribution across relevant frequency bands for speech in all three mask conditions compared to no mask. The differences were not observed in frequencies below 3 kHz. Differences in signal for higher frequencies led to altered acoustic outcomes including spectral tilt. The masks, however, did not significantly influence listener-perceived intelligibility or acoustic measures of perturbation (e.g., HNR, CPPS). Measures of speech rate were lower for N95 and surgical masks, possibly as speakers compensate when wearing masks to improve intelligibility. It is also possible that speech timing differences were related to how speech boundaries are identified in the analysis scripts (i.e., our timing analysis relied on identification of phoneme/word boundaries via intensity thresholds).

Intelligibility scores varied between raters and between mask condition. Anecdotally, it can be difficult to understand people when they wear a mask (Goldin *et al.*, 2020). Our small dataset suggests mask type does not systematically impact intelligibility in controlled environments. Our recordings were made with high-quality microphones in quiet environments. Raters listened to samples in ideal listening conditions away from distractions and background noise but without visual aid (lips and jaw movement) for all mask conditions. In loud environments, communication can be challenging with multiple distractors, background noise, and a lower signal-to-noise ratios (SNRs). Noise in ecological situations may further decrease speech intelligibility, when complementary visual cues blocked by use of face masks play a role in communication. Furthermore, our cohort of subjects included both native and non-native speakers. While our analyses accounted for this, we acknowledge that listeners may have had trouble interpreting accents.

**FIG. 4.**
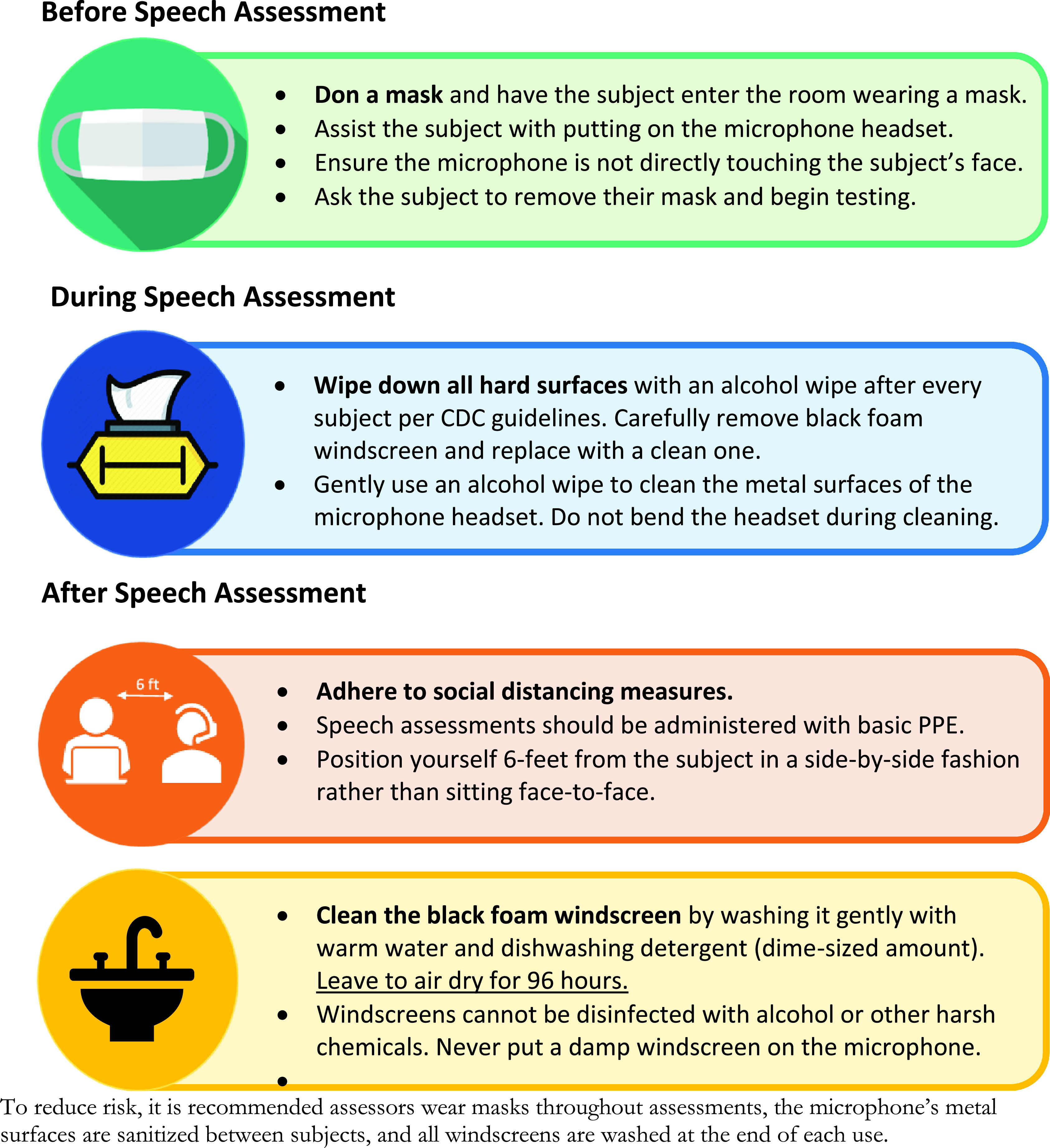
(Color online) Guidance on minimizing risk to patients and staff during speech recordings (reproduced with permission from Redenlab Inc.). *Disclaimer*: Please be advised that nothing completely eliminates bacteria or viruses and the guidelines contained in this document are measures attempting to limit the spread of a virus. Further, these guidelines do not supersede medical practitioner recommendations or the COVID-19 safety policies implemented by your business or institution. It is your responsibility to follow the recommendations and safety policies applicable to your business or institution.*

It is clear that face masks change the acoustic speech signal, but some specific features remain largely unaffected (e.g., acoustic measures of voice quality) irrespective of mask type. These results have implications for clinical assessments and speech research where PPE is required. It is easy to assume that subjects in a speech study will simply remove PPE during assessments; however, subjects and researchers may be reluctant to do so if it leads to potential exposure to airborne viruses. Researchers should consider microphone placement and sound reinforcement systems (e.g., amplified speech signals) in longitudinal studies with data collection requiring PPE throughout the COVID-19 pandemic, to mitigate against changes to protocols that affect speech (see Fig. 4) (Redenlab, 2020).
